# Implementation of controlled quantum teleportation with an arbitrator for secure quantum channels via quantum dots inside optical cavities

**DOI:** 10.1038/s41598-017-14515-5

**Published:** 2017-11-02

**Authors:** Jino Heo, Chang-Ho Hong, Min-Sung Kang, Hyeon Yang, Hyung-Jin Yang, Jong-Phil Hong, Seong-Gon Choi

**Affiliations:** 1 College of Electrical and Computer Engineering, Chungbuk National University, Chungdae-ro 1, Seowon-Gu, Cheongju, Republic of Korea; 2 National Security Research Institute, P.O. Box 1, Yuseong, Daejeon, 34188 Republic of Korea; 3 Center for Quantum Information, Korea Institute of Science and Technology (KIST), Seoul, 136-791 Republic of Korea; 4 Department of Physics, Korea University, Sejong, 339-700 Republic of Korea

## Abstract

We propose a controlled quantum teleportation scheme to teleport an unknown state based on the interactions between flying photons and quantum dots (QDs) confined within single- and double-sided cavities. In our scheme, users (Alice and Bob) can teleport the unknown state through a secure entanglement channel under the control and distribution of an arbitrator (Trent). For construction of the entanglement channel, Trent utilizes the interactions between two photons and the QD-cavity system, which consists of a charged QD (negatively charged exciton) inside a single-sided cavity. Subsequently, Alice can teleport the unknown state of the electron spin in a QD inside a double-sided cavity to Bob’s electron spin in a QD inside a single-sided cavity assisted by the channel information from Trent. Furthermore, our scheme using QD-cavity systems is feasible with high fidelity, and can be experimentally realized with current technologies.

## Introduction

Quantum teleportation (QT) is a quantum information processing scheme^1–18^, which can teleport an unknown state from a sender to a receiver using an entangled state without direct transmission. Since the first QT was proposed by Bennet *et al*.^1^, various QT schemes have been researched and experimentally realized^2–17^. In particular, Karlsson and Bourennane^2^ in 1998 proposed the first controlled quantum teleportation (CQT) using a Greenberg-Horne-Zeilinger (GHZ) state. The essential concept of this CQT scheme is that it is possible for the receiver (Bob) to recover the unknown state with information of the sender (Alice) and also information of a controller (or arbitrator, Trent). After this, CQT schemes were proposed using a Brown state via cavity QED^5^, quantum dots (QDs)^10^, GHZ-like states^12^, nitrogen vacancy centers^13^, a GHZ state in an ion-trapped system^15^, a four-particle cluster state^17^.

For quantum information processing, one of the feasible resources to transfer is a flying photon. And also a photon can be reliably encoded the information due to convenient manipulation by linear optical devices. But this resource exponentially reduces the transmission rate because of optical absorption and noise in the channel (short coherence time). On the other hand, the QD-cavity system^16,19–30^ has received a lot of attention for the storage of quantum information in solid-state quantum systems due to the long electron–spin coherence time (${{\rm{T}}}_{{\rm{2}}}^{{\rm{e}}} \sim {\rm{\mu }}s$)^31–36^ within a limited spin relaxation time (${{\rm{T}}}_{{\rm{2}}}^{{\rm{e}}} \sim {\rm{ms}}$)^37–40^. Furthermore, other research^19,20,40–50^ showed that operations on a single QD spin and preparations for the electron spin state have been developed. Therefore, the various quantum information processing schemes that employ interactions of the photons (fast transferring) and QD-cavity systems (long coherence time), were designed for quantum communications (including quantum teleportation and quantum networks)^16,29,51–59^, quantum controlled operations^24,60–65^, and the analysis and generation of entanglement^21–23,30,66^.

In this paper, we propose a CQT scheme, consisting of an arbitrator (Trent) and two users (Alice and Bob), using the interactions of photons and QDs inside one double-sided and two single-sided optical cavities. Trent, having the QD-cavity system (a single-side cavity)^16,19–22,24,30^, takes the roles of a provider (for the authenticated entanglement channel) and of a controller (for recovering the unknown state). When Trent constructs the entanglement channel, which consists of one electron spin in a QD-cavity system (belonging to Trent) and two photons (transferred to users), Alice and Bob can activate the QT protocol. In the QT process, the unknown state of an electron spin in a QD inside a double-sided cavity^23,25–29^ can be teleported to Bob’s electron spin in a QD inside the single-sided cavity^16,19–24,30^. In addition, Bob requires information from Trent to exactly recover Alice’s unknown state. It means that only an authenticated user via Trent’s distributed entanglement channel can participate in our CQT scheme. To accomplish this CQT scheme, we exploit the interactions between photons and a single electron spin (excess electron) in a single QD inside single-sided^16,19–22,24,30^ and double-sided^23,25–29^ microcavities. After the interactions of the photons and the QD-cavity systems, these generate different reflection and transmission coefficients with phase shifts according to the polarization of photons induced by a single electron spin of a single QD with spin-dependent optical transitions strongly coupled to single-sided^16,19–22,24,30^ and double-sided^23,25–29^ microcavities. Thus, we can acquire the experimental feasibility of our CQT scheme with high fidelity via the QD-cavity systems.

## Interactions between a photon and a singly charged quantum dot inside an optical cavity

For distribution of the authenticated entanglement channel by Trent and the teleportation of the unknown state between Alice and Bob, our scheme utilizes two kinds of QD-cavity system, which consist of a single charged QD inside a resonant micropillar cavity, such as single-^16,19–22,24,30^ and double-sided^23,25–29^ cavities. In this section, we introduce the QD-cavity systems employed in our controlled quantum teleportation scheme.

### A singly charged quantum dot inside a single-sided cavity

**Figure 1:**
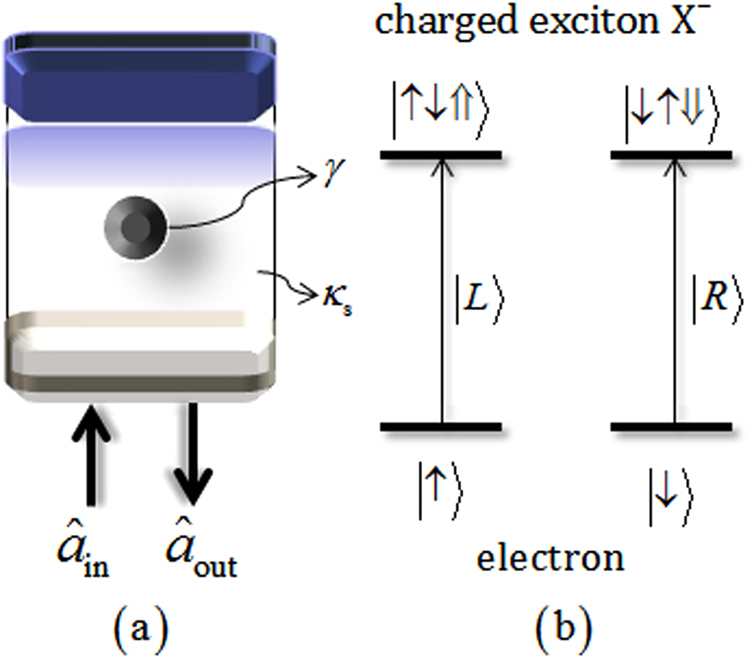
(**a**) A singly charged QD inside a single-sided cavity interacting with a photon where the decay rate (*γ*) of X^−^ and the side leakage rate (*κ*_*s*_). (**b**) The spin selection rule for optical transitions of X^−^ in the QD $|\uparrow \rangle \to |\uparrow \downarrow \Uparrow \rangle$ ($|\downarrow \rangle \to |\downarrow \uparrow \Downarrow \rangle$) is driven by the photon $|L\rangle$ ($|R\rangle$).

A singly charged QD embedded inside a single-sided cavity in Fig. 1(a) ^16,19–22,24,30^ is composed of two GaAs/Al(Ga)As distributed Bragg reflectors (DBRs) and transverse index guiding for three-dimensional confinement of light. The cavity has the bottom DBR partially reflective in terms of incoming and outgoing light of the cavity, while the top DBR is 100% reflective (the single-sided cavity). For maximal light–matter coupling, the QD is located in the center of the single-sided cavity;^16,19–22,24,30^ ${\hat{{a}}}_{{\rm{in}}}$ and ${\hat{{a}}}_{{\rm{out}}}$ are the input and output field operators. Figure 1(b) shows the spin selection rule for spin-dependent optical transitions of a negatively charged exciton (X^−^: consisting of two electrons bound to one hole^67^). Due to the Pauli exclusion principle, the spin-dependent optical transitions are as follows: when the left circularly polarized photon $|L\rangle$ (right circularly polarized photon $|R\rangle$) is injected into the single-sided cavity, the spin state $|\uparrow \rangle \equiv |+1/2\rangle$ ($|\downarrow \rangle \equiv |-1/2\rangle$) of the excess electron can be coupled to X^−^ in the spin state $|\uparrow \downarrow \Uparrow \rangle$ ($|\downarrow \uparrow \Downarrow \rangle$), where $|\Uparrow \rangle$ and $|\Downarrow \rangle$ (*J*_*Z*_ = +3/2 and −3/2) represent heavy-hole spin states. These spin-dependent optical transitions, according to the distinguishable interaction between the polarized photons and the spin states of an electron inside a single-sided cavity, show that the polarized photon can be coupled with the electron spin (hot cavity: $|L\rangle ,\,|\uparrow \rangle$ or $|R\rangle ,\,|\downarrow \rangle$) or not (cold cavity: $|R\rangle ,\,|\uparrow \rangle$ or $|L\rangle ,\,|\downarrow \rangle$). And then, they can induce the different phases and amplitudes after the photons are reflected from the QD-cavity system. The reflection coefficient of this process can be represented via the Heisenberg equation of motion for a cavity field operator $(\hat{{a}})$, a dipole operator $({\hat{\sigma }}_{-})$ of X^−^, and the input-output relations, as follows^68^:

1 $$\begin{array}{rcl}\frac{d\hat{{a}}}{dt} & = & -[i({\omega }_{c}-\omega )+\frac{\kappa }{2}+\frac{{\kappa }_{s}}{2}]\hat{{a}}-g{\hat{\sigma }}_{-}-\sqrt{\kappa }{\hat{{a}}}_{{\rm{in}}},\\ \frac{d{\hat{\sigma }}_{-}}{dt} & = & -[i({\omega }_{{{\rm{X}}}^{-}}-\omega )+\frac{\gamma }{2}]{\hat{\sigma }}_{-}-g{\hat{\sigma }}_{Z}\hat{{a}},\\ {\hat{{a}}}_{{\rm{out}}} & = & {\hat{{a}}}_{{\rm{in}}}+\sqrt{\kappa }\hat{{a}},\end{array}$$

where the frequencies are the external field (*ω*), cavity mode (*ω*_*c*_), and the dipole transition of X^−^ (${\omega }_{{{\rm{X}}}^{-}}$); *g* is the coupling strength between X^−^ and the cavity mode, the cavity field decay rate (*κ*/2), the side leakage rate (*κ*_*s*_/2), and the decay rate (*γ*/2) of X^−^. In the approximation of weak excitation, we take $\langle {\hat{\sigma }}_{Z}\rangle \approx -1$, ${\hat{\sigma }}_{Z}\hat{a}=-\hat{a}$, where the charged QD is in the ground state. And then, the reflection coefficients in the steady state are given by

2 $$\begin{array}{ccc}\frac{{\hat{a}}_{{\rm{o}}{\rm{u}}{\rm{t}}}}{{\hat{a}}_{{\rm{i}}{\rm{n}}}} & = & r(\omega )\Rightarrow \\ {r}_{{\rm{h}}}(\omega )\equiv |{r}_{{\rm{h}}}(\omega )|{e}^{i{\phi }_{{\rm{r}}{\rm{h}}}(\omega )} & = & \frac{[i({\omega }_{c}-\omega )+\gamma /2][i({\omega }_{c}-\omega )-\kappa /2+{\kappa }_{s}/2]+{g}^{2}}{[i({\omega }_{c}-\omega )+\gamma /2][i({\omega }_{c}-\omega )+\kappa /2+{\kappa }_{s}/2]+{g}^{2}},\\ {r}_{0}(\omega )\equiv |{r}_{0}(\omega )|{e}^{i{\phi }_{{\rm{r}}0}(\omega )} & = & \frac{i({\omega }_{c}-\omega )-\kappa /2+{\kappa }_{s}/2}{i({\omega }_{c}-\omega )+\kappa /2+{\kappa }_{s}/2},\end{array}$$

where the dipole of X^−^ is tuned to cavity mode (${\omega }_{{{\rm{X}}}^{-}}={\omega }_{c}$)^16,19–22,24,30^; ${r}_{{\rm{h}}}(\omega )$ is the reflection coefficient when *g* ≠ 0 (hot cavity: the coupled QD with a cavity), and ${r}_{{\rm{0}}}(\omega )$ is the reflection coefficient when *g* = 0 (cold cavity: the uncoupled QD with a cavity). Thus, we can obtain that the reflectance and the phase shift are $|{r}_{{\rm{h}}}(\omega )|$, ${\phi }_{{\rm{rh}}}(\omega )=\text{arg}[{r}_{{\rm{h}}}(\omega )]$ (hot cavity) and $|{r}_{{\rm{0}}}(\omega )|$, ${\phi }_{{\rm{r0}}}(\omega )=\text{arg}[{r}_{{\rm{0}}}(\omega )]$ (cold cavity). Thus, reflection operator $\hat{r}(\omega )$ of the state (photon-spin) after reflection from the QD-cavity system is given by

3 $$\begin{array}{c}\hat{r}(\omega )=|{r}_{0}(\omega )|{e}^{i{\phi }_{{\rm{r0}}}(\omega )}(|R\rangle \langle R|\otimes |\uparrow \rangle \langle \uparrow |+|L\rangle \langle L|\otimes |\downarrow \rangle \langle \downarrow |)\\ \quad \quad \quad +|{r}_{{\rm{h}}}(\omega )|{e}^{i{\phi }_{{\rm{rh}}}(\omega )}(|R\rangle \langle R|\otimes |\downarrow \rangle \langle \downarrow |+|L\rangle \langle L|\otimes |\uparrow \rangle \langle \uparrow |).\end{array}$$

**Figure 2:**
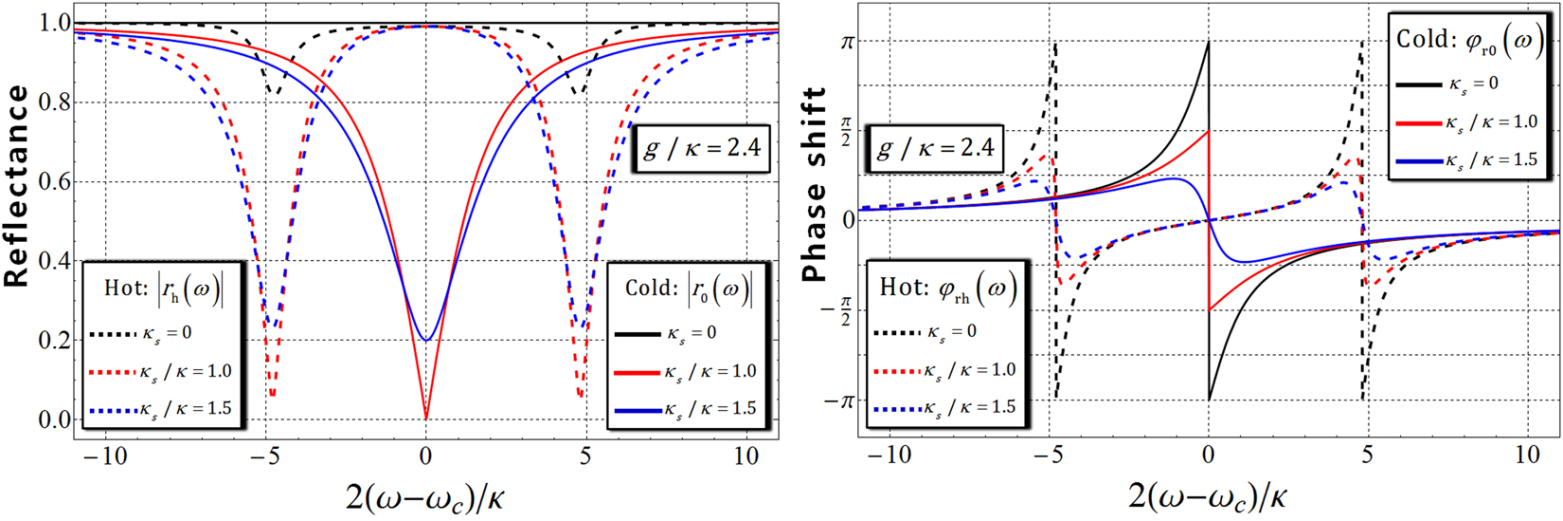
The left figure represents the reflectances, $|{r}_{{\rm{h}}}(\omega )|$ (hot cavity) and $|{r}_{0}(\omega )|$ (cold cavity), and the right figure represents the phase shifts ${\phi }_{{\rm{rh}}}(\omega )$ (hot cavity) and ${\phi }_{{\rm{r0}}}(\omega )$ (cold cavity) for frequency detuning $2(\omega -{\omega }_{c})/\kappa$, according to the difference in the side leakage rates (${\kappa }_{s}=0,{\kappa }_{s}=1.0\kappa \,{\rm{a}}{\rm{n}}{\rm{d}}\,{\kappa }_{s}=1.5\kappa$). $g/\kappa =2.4$ and $\gamma /\kappa =0.1(g > (\kappa ,\gamma ))$ of the practical QD-cavity parameters are taken, and ${\omega }_{{{\rm{X}}}^{-}}={\omega }_{c}$ ^16,19–22,24,30^.

When we take the experimental parameters of the QD-cavity system as $g/\kappa =2.4$ and $\gamma /\kappa =0.1$ $(g > (\kappa ,\gamma ))$ for small *γ* (about several *μ*eV)^19,20,42,69,70^, the reflectances and the phase shifts $[|{r}_{{\rm{h}}}(\omega )|,{\phi }_{{\rm{rh}}}(\omega ):\mathrm{hot\; cavity}]$ and $[|{r}_{0}(\omega )|,{\phi }_{{\rm{r}}0}(\omega ):\mathrm{cold\; cavity}]$ are presented for frequency detuning $2(\omega -{\omega }_{c})/\kappa$ according to the difference in side leakage rates *κ*_*s*_ = 0, *κ*_*s*_ = 1.0 *κ*, and *κ*_*s*_ = 1.5 *κ*, as shown in Fig. 2.

If *κ*_*s*_ is negligible (${\kappa }_{s}\ll \kappa$) with ${\omega }_{{{\rm{X}}}^{-}}={\omega }_{c}$ and frequency detuning $\omega -{\omega }_{c}=\pm \kappa /2$ by adjusting the frequencies of the external field and cavity mode^16,19–22,24,30^, we can get $|{r}_{{\rm{0}}}(\omega )|=|{r}_{{\rm{h}}}(\omega )|\approx 1$, ${\phi }_{{\rm{rh}}}(\omega )=0$, and ${\phi }_{{\rm{r0}}}(\omega )=\mp \pi /2$, as shown in Fig. 2. Thus, if we choose the coupling strength as $g/\kappa =2.4$, $\gamma /\kappa =0.1$, $\omega -{\omega }_{c}=\kappa /2$, and *κ*_*s*_ = 0 (negligible), the reflection operator in Eq. 3 may be described as follows:

4 $$\hat{r}\approx -i|R\rangle \langle R|\otimes |\uparrow \rangle \langle \uparrow |-i|L\rangle \langle L|\otimes |\downarrow \rangle \langle \downarrow |+|R\rangle \langle R|\otimes |\downarrow \rangle \langle \downarrow |+|L\rangle \langle L|\otimes |\uparrow \rangle \langle \uparrow |.$$

Consequently, the reflected photon-spin state from the QD-cavity (single-sided) system can be given by

5 $$|R\rangle |\uparrow \rangle \,\to \,-i|R\rangle |\uparrow \rangle ,\,|L\rangle |\uparrow \rangle \,\to \,|L\rangle |\uparrow \rangle ,\,|R\rangle |\downarrow \rangle \,\to \,|R\rangle |\downarrow \rangle ,\,|L\rangle |\downarrow \rangle \,\to \,-i|L\rangle |\downarrow \rangle ,$$

where $$|{r}_{{\rm{0}}}(\omega )|=|{r}_{{\rm{h}}}(\omega )|\approx 1$$, $${\phi }_{{\rm{rh}}}(\omega )=0$$, and $${\phi }_{{\rm{r0}}}(\omega )=-\pi /2$$ ^16,19–22,24,30^.

### A singly charged quantum dot inside a double-sided cavity

**Figure 3:**
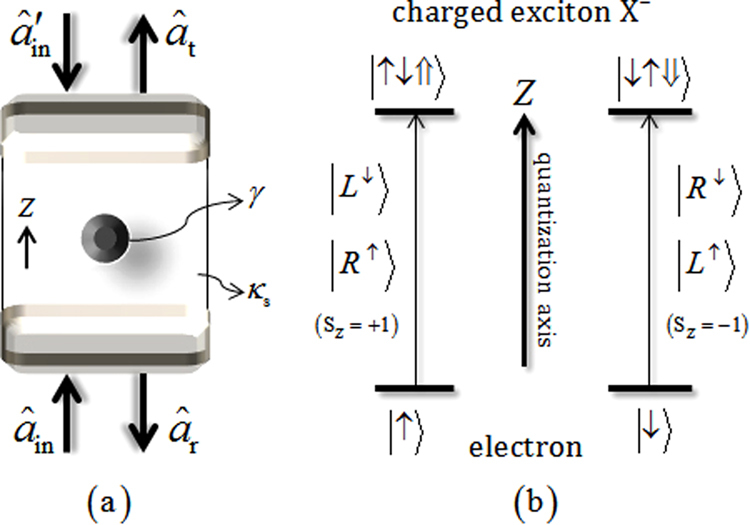
(**a**) A singly charged QD inside a double-sided cavity interacting with a photon where the decay rate (*γ*) of X^−^ and the side leakage rate (*κ*_*s*_). (**b**) The spin selection rule for optical transitions of X^−^ in the QD $|\,\uparrow \rangle \to |\uparrow \downarrow \Uparrow \rangle$ ($|\,\downarrow \rangle \to |\downarrow \uparrow \Downarrow \rangle$) is driven by the photon $|{L}^{\downarrow }\rangle ,|{R}^{\uparrow }\rangle$ ($|{R}^{\downarrow }\rangle ,|{L}^{\uparrow }\rangle$). The symbol $\uparrow$ ($\downarrow$) of photon represents the spin projection along (against) the quantization axis (z direction).

Let us consider a singly charged QD (for a self-assembled InAs/GaAs QD) embedded inside the double-sided cavity in Fig. 3(a) ^23,25–29^. Both the top and bottom DBRs are partially reflective (double-sided cavity); and ${\hat{a}}_{in}$ and ${\hat{a}}_{t}$ (${\hat{a}^{\prime} }_{in}$ and ${\hat{a}}_{r}$) are the input and output field operators along (against) the quantization axis. In Fig. 3(b), when the quantization axis for angular momentum is the z axis for the QD, the spin-dependent optical transitions, due to the Pauli exclusion principle, are as follows: if the polarized photon with respect to the direction of the propagation is $|{R}^{\uparrow }\rangle$ or $|{L}^{\downarrow }\rangle$, ${S}_{z}=+1$, ($|{L}^{\uparrow }\rangle$ or $|{R}^{\downarrow }\rangle$, ${S}_{z}=-1$), the spin state $|\uparrow \rangle$ ($|\downarrow \rangle$) of the excess electron can be coupled to X^−^ in the spin state $|\uparrow \downarrow \Uparrow \rangle$ ($|\downarrow \uparrow \Downarrow \rangle$) – the photon feels a hot cavity ($|{R}^{\uparrow }\rangle |\uparrow \rangle$ or $|{L}^{\downarrow }\rangle |\uparrow \rangle$ or $|{L}^{\uparrow }\rangle |\downarrow \rangle$ or $|{R}^{\downarrow }\rangle |\downarrow \rangle$). While the spin state $|\downarrow \rangle$ ($|\uparrow \rangle$) of the excess electron can be uncoupled, the photon feels a cold cavity ($|{R}^{\uparrow }\rangle |\downarrow \rangle$ or $|{L}^{\downarrow }\rangle |\downarrow \rangle$ or $|{L}^{\uparrow }\rangle |\uparrow \rangle$ or $|{R}^{\downarrow }\rangle |\uparrow \rangle$)^23,25–29^. According to the hot or cold cavity of the spin-dependent optical transitions, the difference in the reflection and transmission coefficients of this QD-cavity system (double-sided) can be acquired by solving the Heisenberg equation of motion for cavity field operator $(\hat{a})$, the dipole operator $({\hat{\sigma }}_{-})$ of X^−^, and the input-output relations, as follows^68^:

6 $$\begin{array}{ccc}\frac{d\hat{{a}}}{dt} & = & -[i({\omega }_{c}-\omega )+\kappa +\frac{{\kappa }_{s}}{2}]\hat{{a}}-g{\hat{{\sigma }}}_{-}-\sqrt{\kappa }({\hat{{a}}}_{{\rm{i}}{\rm{n}}}+{\hat{{a}}}_{{\rm{i}}{\rm{n}}}^{^{\prime} }),\\ \frac{d{\hat{{\sigma }}}_{-}}{dt} & = & -[i({\omega }_{{{\rm{X}}}^{-}}-\omega )+\frac{\gamma }{2}]{\hat{{\sigma }}}_{-}-g{\hat{{\sigma }}}_{Z}\hat{{a}},\\ {\hat{{a}}}_{{\rm{r}}} & = & {\hat{{a}}}_{{\rm{i}}{\rm{n}}}+\sqrt{\kappa }\hat{{a}},\\ {\hat{{a}}}_{{\rm{t}}} & = & {\hat{{a}}}_{{\rm{i}}{\rm{n}}}^{^{\prime} }+\sqrt{\kappa }\hat{{a}}.\end{array}$$

In the approximation of weak excitation, we take $\langle {\hat{\sigma }}_{Z}\rangle \approx -1$, ${\hat{{\sigma }}}_{Z}\hat{{a}}=-\hat{{a}}$, where the charged QD is in the ground state. And then, the reflection and transmission coefficients in the steady state are given by

7 $$\begin{array}{rcl}\frac{{\hat{{a}}}_{{\rm{r}}}}{{\hat{{a}}}_{{\rm{in}}}}=\frac{{\hat{{a}}}_{{\rm{t}}}}{{\hat{{a}}}_{{\rm{in}}}^{{^{\prime} }}} & = & R(\omega )\,\Rightarrow \\ {R}_{{\rm{h}}}(\omega )\equiv |{R}_{{\rm{h}}}(\omega )|{e}^{i{\phi }_{{\rm{Rh}}}(\omega )} & = & \frac{[i({\omega }_{c}-\omega )+\gamma /2][i({\omega }_{c}-\omega )+{\kappa }_{s}/2]+{g}^{2}}{[i({\omega }_{c}-\omega )+\gamma /2][i({\omega }_{c}-\omega )+\kappa +{\kappa }_{s}/2]+{g}^{2}},\\ {R}_{0}(\omega )\equiv |{R}_{0}(\omega )|{e}^{i{\phi }_{{\rm{R0}}}(\omega )} & = & \frac{i({\omega }_{c}-\omega )+{\kappa }_{s}/2}{i({\omega }_{c}-\omega )+\kappa +{\kappa }_{s}/2},\\ \frac{{\hat{{a}}}_{{\rm{t}}}}{{\hat{{a}}}_{{\rm{in}}}}=\frac{{\hat{{a}}}_{{\rm{r}}}}{{\hat{{a}}}_{{\rm{in}}}^{{^{\prime} }}} & = & R(\omega )-1=T(\omega )\,\Rightarrow \\ {T}_{{\rm{h}}}(\omega )\equiv |{T}_{{\rm{h}}}(\omega )|{e}^{i{\phi }_{{\rm{Th}}}(\omega )} & = & \frac{-\kappa [i({\omega }_{c}-\omega )+\gamma /2]}{[i({\omega }_{c}-\omega )+\gamma /2][i({\omega }_{c}-\omega )+\kappa +{\kappa }_{s}/2]+{g}^{2}},\\ {T}_{0}(\omega )\equiv |{T}_{0}(\omega )|{e}^{i{\phi }_{{\rm{T0}}}(\omega )} & = & \frac{-\kappa }{i({\omega }_{c}-\omega )+\kappa +{\kappa }_{s}/2},\end{array}$$

where ${\omega }_{{{\rm{X}}}^{-}}={\omega }_{c}$ for simplification^23,25–29^. ${R}_{{\rm{h}}}(\omega )$ $({T}_{{\rm{h}}}(\omega ))$ is the reflection (transmission) coefficient when $g\ne 0$ (hot cavity: the coupled QD with a cavity), and ${R}_{{\rm{0}}}(\omega )$ $({T}_{{\rm{0}}}(\omega ))$ is the reflection (transmission) coefficient when g = 0 (cold cavity: the uncoupled QD with a cavity). Because of the spin-dependent optical transitions (polarization and direction of the propagation of photon, and spin of electron), the polarization and the propagated direction of the reflected photon from the QD-cavity system will be flipped after the interaction between photon and QD^23,25–29^. Thus, reflection $\hat{R}(\omega )$ and transmission $\hat{T}(\omega )$ operators of the state (photon-spin), after the interaction with the QD-cavity system, are given by

8 $$\begin{array}{c}\hat{R}(\omega )=|{R}_{{\rm{h}}}(\omega )|{e}^{i{\phi }_{{\rm{Rh}}}(\omega )}[(|{R}^{\uparrow }\rangle \langle {L}^{\downarrow }|+|{L}^{\downarrow }\rangle \langle {R}^{\uparrow }|)\otimes |\uparrow \rangle \langle \uparrow |+(|{R}^{\downarrow }\rangle \langle {L}^{\uparrow }|+|{L}^{\uparrow }\rangle \langle {R}^{\downarrow }|)\otimes |\downarrow \rangle \langle \downarrow |]\\ \quad \quad \quad \,+|{R}_{{\rm{0}}}(\omega )|{e}^{i{\phi }_{{\rm{R0}}}(\omega )}[(|{R}^{\downarrow }\rangle \langle {L}^{\uparrow }|+|{L}^{\uparrow }\rangle \langle {R}^{\downarrow }|)\otimes |\uparrow \rangle \langle \uparrow |+(|{R}^{\uparrow }\rangle \langle {L}^{\downarrow }|+|{L}^{\downarrow }\rangle \langle {R}^{\uparrow }|)\otimes |\downarrow \rangle \langle \downarrow |],\\ \hat{T}(\omega )=|{T}_{{\rm{h}}}(\omega )|{e}^{i{\phi }_{{\rm{Th}}}(\omega )}[(|{R}^{\uparrow }\rangle \langle {R}^{\uparrow }|+|{L}^{\downarrow }\rangle \langle {L}^{\downarrow }|)\otimes |\uparrow \rangle \langle \uparrow |+(|{R}^{\downarrow }\rangle \langle {R}^{\downarrow }|+|{L}^{\uparrow }\rangle \langle {L}^{\uparrow }|)\otimes |\downarrow \rangle \langle \downarrow |]\\ \,\quad \quad \quad +|{T}_{{\rm{0}}}(\omega )|{e}^{i{\phi }_{{\rm{T0}}}(\omega )}[(|{R}^{\downarrow }\rangle \langle {R}^{\downarrow }|+|{L}^{\uparrow }\rangle \langle {L}^{\uparrow }|)\otimes |\uparrow \rangle \langle \uparrow |+(|{R}^{\uparrow }\rangle \langle {R}^{\uparrow }|+|{L}^{\downarrow }\rangle \langle {L}^{\downarrow }|)\otimes |\downarrow \rangle \langle \downarrow |].\end{array}$$

**Figure 4:**
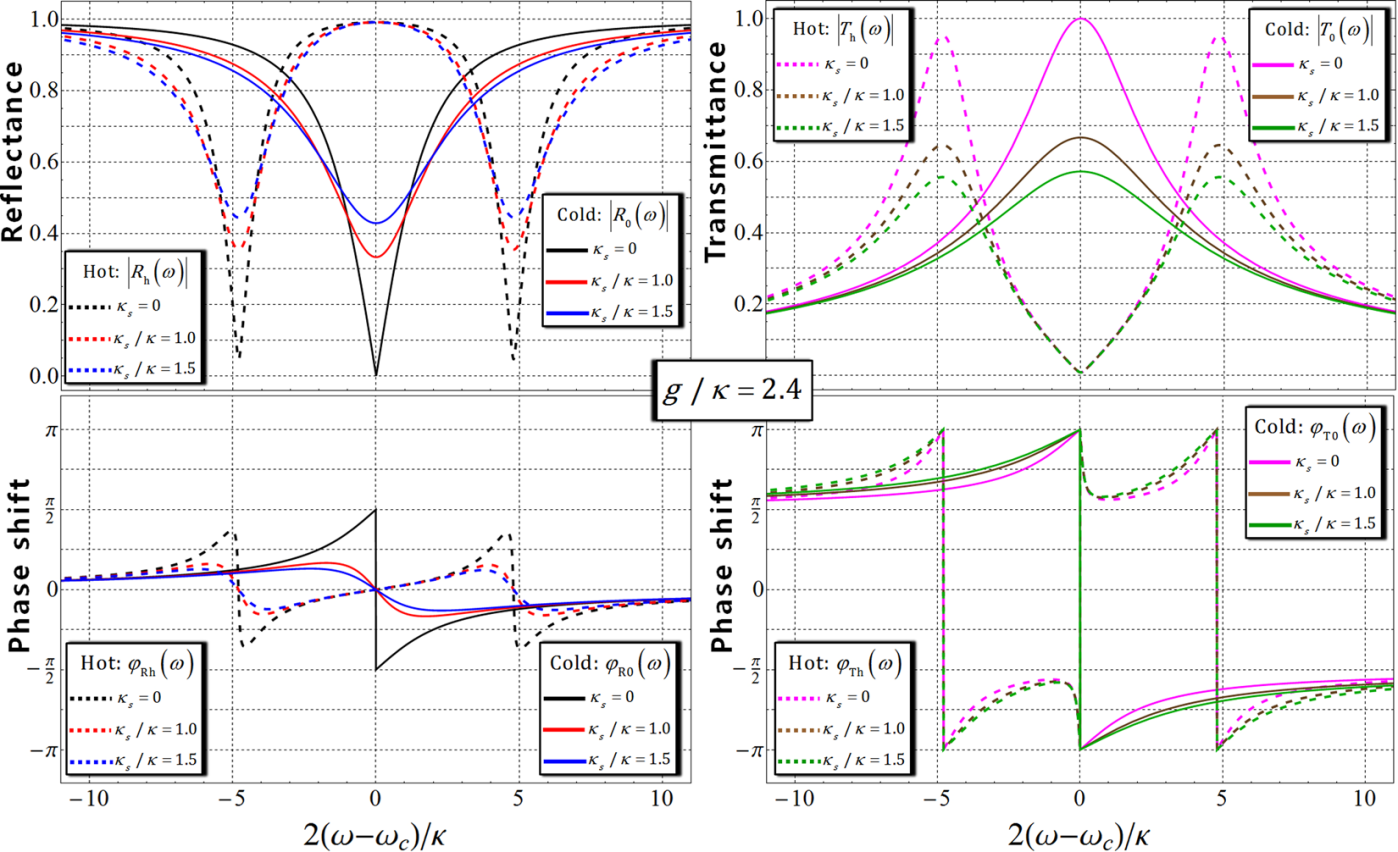
The two left figures represent the reflectances and phase shifts for $(|{R}_{{\rm{h}}}(\omega )|,\,{\phi }_{{\rm{Rh}}}(\omega ):\mathrm{hot}\,{\rm{cavity}})$ and $(|{R}_{{\rm{0}}}(\omega )|,{\phi }_{{\rm{R0}}}(\omega ):\mathrm{cold}\,{\rm{cavity}})$; in addition, $(|{T}_{{\rm{h}}}(\omega )|,{\phi }_{{\rm{Th}}}(\omega ):\mathrm{hot\; cavity})$ and $(|{T}_{{\rm{0}}}(\omega )|,\,{\phi }_{{\rm{T0}}}(\omega ):\mathrm{cold\; cavity})$ are represented in the two right figures for frequency detuning $2(\omega -{\omega }_{c})/\kappa$, according to the difference in the side leakage rates (${\kappa }_{s}=0,{\kappa }_{s}=1.0\kappa {\rm{a}}{\rm{n}}{\rm{d}}\,{\kappa }_{s}=1.5\kappa$); $g/\kappa =2.4$ and $\gamma /\kappa =0.1(g > (\kappa ,\gamma ))$ of the practical QD-cavity parameters are taken, and ${\omega }_{{{\rm{X}}}^{-}}={\omega }_{c}$ ^23,25–29^.

If the experimental parameters of the QD-cavity system are taken as $g/\kappa =2.4$ and $\gamma /\kappa =0.1$ $(g > (\kappa ,\,\gamma ))$ with small *γ* (about several *μ*eV)^19,20,42,69,70^, the reflectances, the transmittances, and the phase shifts as $[|{R}_{{\rm{h}}}(\omega )|,\,{\phi }_{{\rm{Rh}}}(\omega ),\,|{T}_{{\rm{h}}}(\omega )|,\,{\phi }_{{\rm{Th}}}(\omega ):\,{\rm{hot}}\,{\rm{cavity}}]$ and $[|{R}_{0}(\omega )|,\,{\phi }_{{\rm{R}}0}(\omega ),\,|{T}_{0}(\omega )|,\,{\phi }_{{\rm{T}}0}(\omega ):\,{\rm{c}}{\rm{o}}{\rm{l}}{\rm{d}}\,{\rm{c}}{\rm{a}}{\rm{v}}{\rm{i}}{\rm{t}}{\rm{y}}]$ can be plotted for frequency detuning $2(\omega -{\omega }_{c})/\kappa$ according to the difference in the side leakage rates *κ*_*s*_ = 0, *κ*_*s*_ *=* 1.0 *κ*, and *κ*_*s*_ *=* 1.5 *κ*, as shown in Fig. 4.

If *κ*_*s*_ is negligible (${\kappa }_{s}\ll \kappa$) with ${\omega }_{{{\rm{X}}}^{-}}={\omega }_{c}$, and frequency detuning $\omega ={\omega }_{c}$ by adjusting the frequencies of the external field and cavity mode in the resonant condition^23,25–29^, we can get $|{R}_{{\rm{h}}}(\omega )|=|{T}_{{\rm{0}}}(\omega )|\approx 1$, $|{R}_{{\rm{0}}}(\omega )|=|{T}_{{\rm{h}}}(\omega )|\approx 0$, ${\phi }_{{\rm{R0}}}(\omega )={\phi }_{{\rm{Rh}}}(\omega )\approx 0$, and ${\phi }_{{\rm{T0}}}(\omega )={\phi }_{{\rm{Th}}}(\omega )\approx \pi$, as shown in Fig. 4. Thus, if we choose the coupling strength as $g/\kappa =2.4$, $\gamma /\kappa =0.1$, $\omega ={\omega }_{c}$, and *κ*_*s*_ = 0 (negligible), the reflection and transmission operators in Eq. 8 may be described as follows:

9 $$\begin{array}{rcl}\hat{R} & \approx  & (|{R}^{\uparrow }\rangle \langle {L}^{\downarrow }|+|{L}^{\downarrow }\rangle \langle {R}^{\uparrow }|)\otimes |\uparrow \rangle \langle \uparrow |+(|{R}^{\downarrow }\rangle \langle {L}^{\uparrow }|+|{L}^{\uparrow }\rangle \langle {R}^{\downarrow }|)\otimes |\downarrow \rangle \langle \downarrow |\\ \hat{T} & \approx  & -(|{R}^{\downarrow }\rangle \langle {R}^{\downarrow }|+|{L}^{\uparrow }\rangle \langle {L}^{\uparrow }|)\otimes |\uparrow \rangle \langle \uparrow |-(|{R}^{\uparrow }\rangle \langle {R}^{\uparrow }|+|{L}^{\downarrow }\rangle \langle {L}^{\downarrow }|)\otimes |\downarrow \rangle \langle \downarrow |.\end{array}$$

Consequently, the reflected or transmitted photon-spin state from the QD-cavity system (double-sided) can be given by

10 $$\begin{array}{ccc}|{R}^{\uparrow }\rangle |\uparrow \rangle  & \to  & |{L}^{\downarrow }\rangle |\uparrow \rangle ,\,\,\,|{L}^{\downarrow }\rangle |\uparrow \rangle \,\to \,|{R}^{\uparrow }\rangle |\uparrow \rangle ,\\ |{R}^{\downarrow }\rangle |\downarrow \rangle  & \to  & |{L}^{\uparrow }\rangle |\downarrow \rangle ,\,\,\,|{L}^{\uparrow }\rangle |\downarrow \rangle \,\to \,|{R}^{\downarrow }\rangle |\downarrow \rangle ,\,\,\,({\rm{r}}{\rm{e}}{\rm{f}}{\rm{l}}{\rm{e}}{\rm{c}}{\rm{t}}{\rm{i}}{\rm{o}}{\rm{n}})\\ |{R}^{\downarrow }\rangle |\uparrow \rangle  & \to  & -|{R}^{\downarrow }\rangle |\uparrow \rangle ,\,|{L}^{\uparrow }\rangle |\uparrow \rangle \,\to \,-|{L}^{\uparrow }\rangle |\uparrow \rangle ,\\ |{R}^{\uparrow }\rangle |\downarrow \rangle  & \to  & -|{R}^{\uparrow }\rangle |\downarrow \rangle ,\,|{L}^{\downarrow }\rangle |\downarrow \rangle \,\to \,-|{L}^{\downarrow }\rangle |\downarrow \rangle ,({\rm{t}}{\rm{r}}{\rm{a}}{\rm{n}}{\rm{s}}{\rm{m}}{\rm{i}}{\rm{s}}{\rm{s}}{\rm{i}}{\rm{o}}{\rm{n}})\end{array}$$

where $$|{R}_{{\rm{h}}}(\omega )|=|{T}_{{\rm{0}}}(\omega )|\approx 1$$, $$|{R}_{{\rm{0}}}(\omega )|=|{T}_{{\rm{h}}}(\omega )|\approx 0$$, $${\phi }_{{\rm{R0}}}(\omega )={\phi }_{{\rm{Rh}}}(\omega )\approx 0$$, and $${\phi }_{{\rm{T0}}}(\omega )={\phi }_{{\rm{Th}}}(\omega )\approx \pi $$ ^23,25–29^.

## Controlled quantum teleportation with an arbitrator via quantum dots inside single- and double-sided cavities

**Figure 5:**
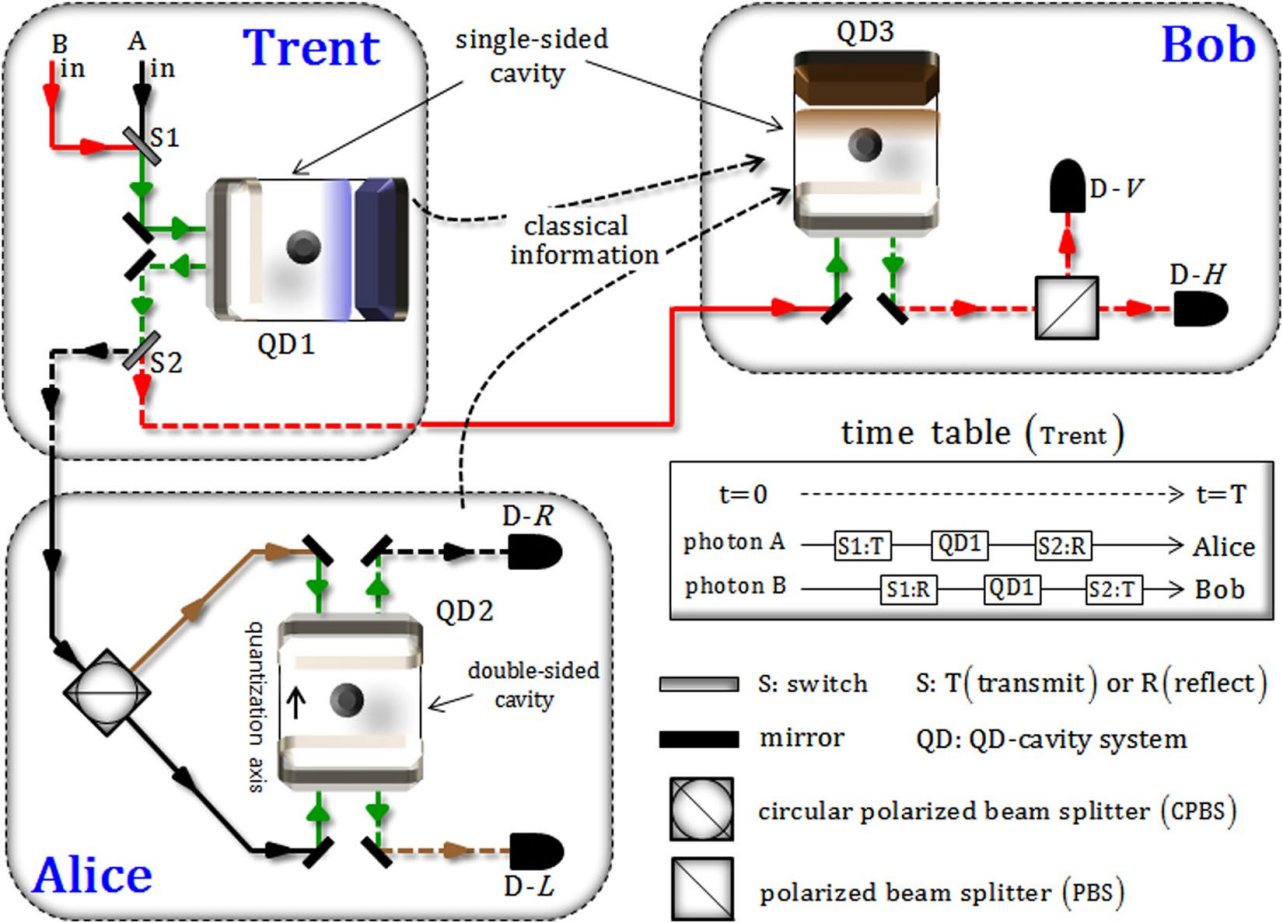
In our proposed CQT scheme, Trent provides an entanglement channel to Alice and Bob using the interactions between two photons and the single-sided QD-cavity system (QD1). Then, Alice can teleport the unknown state of an electron in the double-sided QD-cavity system (QD2) to Bob through the entanglement channel. Subsequently, for reconstruction of a teleported unknown state in the single-sided QD-cavity system (QD3) on Bob’s side, the classical information of Trent and Alice are required. Here, the resulting measurement of the electron spin of QD1 (Trent) guarantees authentication of the entanglement channel for secure communication.

We propose a controlled quantum teleportation scheme in which an arbitrator (Trent) and two users (Alice and Bob) utilize the interactions of the photons and the QDs inside one double-sided^23,25–29^ and two single-sided^16,19–22,24,30^ optical cavities. Our CQT scheme consists of a channel provider [Trent, having the single-sided QD-cavity system (QD1)], a sender [Alice, having the double-sided QD-cavity system (QD2)], and a receiver [Bob, having a single-sided QD-cavity system (QD3)], as illustrated in Fig. 5.

First, Trent distributes an entanglement channel (two photonic spins A, B, and electron spin 1 in QD1) via the interactions of two photons and the QD-cavity system (single-sided), to teleport an unknown state between users. In Fig. 5, let us assume that Trent prepares the initial state as ${|{-}_{{\rm{s}}}\rangle }_{1}\otimes {|H\rangle }_{{\rm{A}}}\otimes {|H\rangle }_{{\rm{B}}}$ of the photon–electron system. Here, we define the relations of the spin state of an excess electron in QD and the polarizations of a photon as follows:

11 $$\begin{array}{c}|{\pm }_{{\rm{s}}}\rangle \equiv (|\uparrow \rangle \pm |\downarrow \rangle )/\sqrt{2},\quad \quad |\pm {{\rm{y}}}_{{\rm{s}}}\rangle \equiv (|\uparrow \rangle \pm i|\downarrow \rangle )/\sqrt{2},\\ |H\rangle \equiv (|R\rangle +|L\rangle )/\sqrt{2},\quad \quad |V\rangle \equiv (|R\rangle -|L\rangle )/\sqrt{2},\end{array}$$

where $|H\rangle$ (horizontal) and $|V\rangle$ (vertical) represent the linearly polarized photon. After the interactions between photons (A and B) and QD1 due to the time table in Fig. 5 in sequence, the initial state will be transformed to ${|{{\boldsymbol{\phi }}}_{{\rm{t}}}\rangle }_{1{\rm{AB}}}$ (three-qubit entangled state) according to Eq. 5 (the interaction of a single-sided cavity)^16,19–22,24,30^:

12 $$\begin{array}{rcl}{|{{\boldsymbol{\phi }}}_{{\rm{t}}}\rangle }_{1{\rm{AB}}} & = & \frac{-1}{\sqrt{2}}[{|\uparrow \rangle }_{{\rm{1}}}\otimes \frac{1}{\sqrt{2}}({|R\rangle }_{{\rm{A}}}+i{|L\rangle }_{{\rm{A}}})\frac{1}{\sqrt{2}}({|R\rangle }_{{\rm{B}}}+i{|L\rangle }_{{\rm{B}}})\\  &  & -\,{|\downarrow \rangle }_{{\rm{1}}}\otimes \frac{1}{\sqrt{2}}({|R\rangle }_{{\rm{A}}}-i{|L\rangle }_{{\rm{A}}})\frac{1}{\sqrt{2}}({|R\rangle }_{{\rm{B}}}-i{|L\rangle }_{{\rm{B}}})]\\  & = & \frac{-i}{\sqrt{2}}{|{+}_{{\rm{s}}}\rangle }_{1}\otimes \frac{1}{\sqrt{2}}({|R\rangle }_{{\rm{A}}}{|L\rangle }_{{\rm{B}}}+{|L\rangle }_{{\rm{A}}}{|R\rangle }_{{\rm{B}}})\\  &  & +\,\frac{-1}{\sqrt{2}}{|{-}_{{\rm{s}}}\rangle }_{1}\otimes \frac{1}{\sqrt{2}}({|R\rangle }_{{\rm{A}}}{|R\rangle }_{{\rm{B}}}-{|L\rangle }_{{\rm{A}}}{|L\rangle }_{{\rm{B}}}).\end{array}$$

Subsequently, Trent sends two photons, A and B, to Alice and Bob, respectively, and electron spin 1 remains (is stored) in QD1 on Trent’s side.

On Alice’s side, secondly, the unknown state, $\alpha {|\uparrow \rangle }_{{\rm{2}}}+\beta {|\downarrow \rangle }_{{\rm{2}}}$, of electron spin 2 inside a double-sided cavity (QD2), as in Fig. 5, can be prepared^19,20,40–50^. Then, Alice employs the interaction (Eq. 9) between photon A (transferred from Trent) and QD2 (electron spin 2 inside a double-sided cavity)^23,25–29^ to teleport the unknown state to Bob after photon A passes through the circular polarized beam splitter (CPBS). Thus, the state of ${|{{\boldsymbol{\phi }}}_{{\rm{t}}}\rangle }_{1{\rm{AB}}}$, Eq. 12, will be transformed to the output state, ${|{{\boldsymbol{\phi }}}_{{\rm{A}}}\rangle }_{1{\rm{A2B}}}$, of the photons (A and B) and electrons (1 and 2), as follows:

13 $$\begin{array}{c}{|{{\boldsymbol{\phi }}}_{{\rm{A}}}\rangle }_{1{\rm{A2B}}}=\frac{-i}{2}{|{+}_{{\rm{s}}}\rangle }_{1}\otimes [{|{R}^{\uparrow }\rangle }_{{\rm{A}}}(\alpha {|\uparrow \rangle }_{{\rm{2}}}{|R\rangle }_{{\rm{B}}}-\beta {|\downarrow \rangle }_{{\rm{2}}}{|L\rangle }_{{\rm{B}}})+{|{L}^{\downarrow }\rangle }_{{\rm{A}}}(\alpha {|\uparrow \rangle }_{{\rm{2}}}{|L\rangle }_{{\rm{B}}}-\beta {|\downarrow \rangle }_{{\rm{2}}}{|R\rangle }_{{\rm{B}}})]\\ \quad \,\,\quad \quad \quad +\frac{1}{2}{|{-}_{{\rm{s}}}\rangle }_{1}\otimes [{|{R}^{\uparrow }\rangle }_{{\rm{A}}}(\alpha {|\uparrow \rangle }_{{\rm{2}}}{|L\rangle }_{{\rm{B}}}+\beta {|\downarrow \rangle }_{{\rm{2}}}{|R\rangle }_{{\rm{B}}})-{|{L}^{\downarrow }\rangle }_{{\rm{A}}}(\alpha {|\uparrow \rangle }_{{\rm{2}}}{|R\rangle }_{{\rm{B}}}+\beta {|\downarrow \rangle }_{{\rm{2}}}{|L\rangle }_{{\rm{B}}})],\end{array}$$

where the operation of the CPBS shows that the polarization $|R\rangle (|L\rangle )$ of the photon is transmitted (reflected). Then, photon A, and electron 2 within QD2 remain on Alice’s side.

Third, for the reconstruction of Alice’s unknown state, $\alpha {|\uparrow \rangle }_{{\rm{2}}}+\beta {|\downarrow \rangle }_{{\rm{2}}}$, Bob prepares the state as $({|\uparrow \rangle }_{{\rm{3}}}+{|\downarrow \rangle }_{{\rm{3}}})/\sqrt{2}$ of electron spin 3 in QD3 inside a single-sided cavity. After the interaction between photon B (transferred from Trent) and QD3 (electron spin 3 inside a single-sided cavity) according to Eq. 5 ^16,19–22,24,30^, photon B passes through the polarized beam splitter (PBS), which transmits $|H\rangle$, and reflects $|V\rangle$. Subsequently, the final state, ${|{{\boldsymbol{\phi }}}_{{\rm{B}}}\rangle }_{1{\rm{A2B3}}}$, of the total system (pre-measurement) is given by

14 $$\begin{array}{c}{|{{\boldsymbol{\phi }}}_{{\rm{B}}}\rangle }_{1{\rm{A2B3}}}=\frac{-1}{4}{|{+}_{{\rm{s}}}\rangle }_{1}\otimes [{|{R}^{\uparrow }\rangle }_{{\rm{A}}}{|{+}_{{\rm{s}}}\rangle }_{{\rm{2}}}\otimes \{{|H\rangle }_{{\rm{B}}}(\alpha {|+{{\rm{y}}}_{{\rm{s}}}\rangle }_{{\rm{3}}}-i\beta {|-{{\rm{y}}}_{{\rm{s}}}\rangle }_{{\rm{3}}})+{|V\rangle }_{{\rm{B}}}(\alpha {|+{{\rm{y}}}_{{\rm{s}}}\rangle }_{{\rm{3}}}+i\beta {|-{{\rm{y}}}_{{\rm{s}}}\rangle }_{{\rm{3}}})\}\\ \quad \quad \quad \quad \,\,\,+{|{R}^{\uparrow }\rangle }_{{\rm{A}}}{|{-}_{{\rm{s}}}\rangle }_{{\rm{2}}}\otimes \{{|H\rangle }_{{\rm{B}}}(\alpha {|+{{\rm{y}}}_{{\rm{s}}}\rangle }_{{\rm{3}}}+i\beta {|-{{\rm{y}}}_{{\rm{s}}}\rangle }_{{\rm{3}}})+{|V\rangle }_{{\rm{B}}}(\alpha {|+{{\rm{y}}}_{{\rm{s}}}\rangle }_{{\rm{3}}}-i\beta {|-{{\rm{y}}}_{{\rm{s}}}\rangle }_{{\rm{3}}})\}\\ \quad \quad \quad \quad \,\,\,+i{|{L}^{\downarrow }\rangle }_{{\rm{A}}}{|{+}_{{\rm{s}}}\rangle }_{{\rm{2}}}\otimes \{{|H\rangle }_{{\rm{B}}}(\alpha {|-{{\rm{y}}}_{{\rm{s}}}\rangle }_{{\rm{3}}}+i\beta {|+{{\rm{y}}}_{{\rm{s}}}\rangle }_{{\rm{3}}})-{|V\rangle }_{{\rm{B}}}(\alpha {|-{{\rm{y}}}_{{\rm{s}}}\rangle }_{{\rm{3}}}-i\beta {|+{{\rm{y}}}_{{\rm{s}}}\rangle }_{{\rm{3}}})\}\\ \quad \quad \quad \quad \,\,\,+i{|{L}^{\downarrow }\rangle }_{{\rm{A}}}{|{-}_{{\rm{s}}}\rangle }_{{\rm{2}}}\otimes \{{|H\rangle }_{{\rm{B}}}(\alpha {|-{{\rm{y}}}_{{\rm{s}}}\rangle }_{{\rm{3}}}-i\beta {|+{{\rm{y}}}_{{\rm{s}}}\rangle }_{{\rm{3}}})-{|V\rangle }_{{\rm{B}}}(\alpha {|-{{\rm{y}}}_{{\rm{s}}}\rangle }_{{\rm{3}}}+i\beta {|+{{\rm{y}}}_{{\rm{s}}}\rangle }_{{\rm{3}}})\}]\\ \quad \quad \quad \quad \,\,\,+\frac{1}{4}{|{-}_{{\rm{s}}}\rangle }_{1}\otimes [{|{R}^{\uparrow }\rangle }_{{\rm{A}}}{|{+}_{{\rm{s}}}\rangle }_{{\rm{2}}}\otimes \{{|H\rangle }_{{\rm{B}}}(\alpha {|-{{\rm{y}}}_{{\rm{s}}}\rangle }_{{\rm{3}}}-i\beta {|+{{\rm{y}}}_{{\rm{s}}}\rangle }_{{\rm{3}}})-{|V\rangle }_{{\rm{B}}}(\alpha {|-{{\rm{y}}}_{{\rm{s}}}\rangle }_{{\rm{3}}}+i\beta {|+{{\rm{y}}}_{{\rm{s}}}\rangle }_{{\rm{3}}})\}\\ \quad \quad \quad \quad \,\,\,+{|{R}^{\uparrow }\rangle }_{{\rm{A}}}{|{-}_{{\rm{s}}}\rangle }_{{\rm{2}}}\otimes \{{|H\rangle }_{{\rm{B}}}(\alpha {|-{{\rm{y}}}_{{\rm{s}}}\rangle }_{{\rm{3}}}+i\beta {|+{{\rm{y}}}_{{\rm{s}}}\rangle }_{{\rm{3}}})-{|V\rangle }_{{\rm{B}}}(\alpha {|-{{\rm{y}}}_{{\rm{s}}}\rangle }_{{\rm{3}}}-i\beta {|+{{\rm{y}}}_{{\rm{s}}}\rangle }_{{\rm{3}}})\}\\ \quad \quad \quad \quad \,\,\,+i{|{L}^{\downarrow }\rangle }_{{\rm{A}}}{|{+}_{{\rm{s}}}\rangle }_{{\rm{2}}}\otimes \{{|H\rangle }_{{\rm{B}}}(\alpha {|+{{\rm{y}}}_{{\rm{s}}}\rangle }_{{\rm{3}}}+i\beta {|-{{\rm{y}}}_{{\rm{s}}}\rangle }_{{\rm{3}}})+{|V\rangle }_{{\rm{B}}}(\alpha {|+{{\rm{y}}}_{{\rm{s}}}\rangle }_{{\rm{3}}}-i\beta {|-{{\rm{y}}}_{{\rm{s}}}\rangle }_{{\rm{3}}})\}\\ \quad \quad \quad \quad \,\,\,+i{|{L}^{\downarrow }\rangle }_{{\rm{A}}}{|{-}_{{\rm{s}}}\rangle }_{{\rm{2}}}\otimes \{{|H\rangle }_{{\rm{B}}}(\alpha {|+{{\rm{y}}}_{{\rm{s}}}\rangle }_{{\rm{3}}}-i\beta {|-{{\rm{y}}}_{{\rm{s}}}\rangle }_{{\rm{3}}})+{|V\rangle }_{{\rm{B}}}(\alpha {|+{{\rm{y}}}_{{\rm{s}}}\rangle }_{{\rm{3}}}+i\beta {|-{{\rm{y}}}_{{\rm{s}}}\rangle }_{{\rm{3}}})\}].\end{array}$$

Finally, Trent, Alice, and Bob conduct the measurements. Electron spin 1 in QD1 is measured (basis $\{|{+}_{{\rm{s}}}\rangle ,\,|{-}_{{\rm{s}}}\rangle \}$) by Trent, and Alice measures electron spin 2 (basis $\{|{+}_{{\rm{s}}}\rangle ,\,|{-}_{{\rm{s}}}\rangle \}$) in QD2 and photon A (by photon detector D-*R* or D-*L* in Fig. 5). Bob measures photon B, which passes through the PBS via photon detector D-*H* or D-*V*, as in Fig. 5. According to Eq. 14, Bob requires Alice’s classical information of the measurement results of electron spin 2 and the polarization of photon A as well as Trent’s classical information of the measurement result of electron spin 1 to precisely reconstruct Alice’s unknown state to electron spin 3 of the QD (belonging to Bob) by unitary operations^19,20,40–50^. Table 1 shows all possible states of Bob’s electron spin 3 and the optimal unitary operations due to the classical information (Alice → Bob: two bits) and (Trent → Bob: one bit) after the measurement procedures. Furthermore, the measurement result of electron spin 1 by Trent is the essential information to accomplish the teleportation between users in our CQT scheme. This means that users, who are activating the QT process, can be confirmed as to whether they received the authenticated entanglement channel from Trent in the procedure of the distribution quantum channel or not.

**Table 1:** The teleported states (Alice → Bob) on electron 3 and unitary operations, according to the measurement results of electron spin 1 (Trent), and spin 2 and photon A (Alice).

Trent’s result of electron spin 1	Alice’s results of photon A and electron spin 2	Bob’s result of photon B	Teleported unknown state to electron spin 3	Bob’s unitary operation
${|{+}_{{\rm{S}}}\rangle }_{{\rm{1}}}$	${|R\rangle }_{{\rm{A}}}{|{\pm }_{{\rm{S}}}\rangle }_{2}$	${|H\rangle }_{{\rm{B}}}$	$\alpha {|+{{\rm{y}}}_{{\rm{S}}}\rangle }_{3}\mp i\beta {|-{{\rm{y}}}_{{\rm{S}}}\rangle }_{3}$	$|\uparrow \rangle \langle +{{\rm{y}}}_{{\rm{S}}}|\pm i|\downarrow \rangle \langle -{{\rm{y}}}_{{\rm{S}}}|$
		${|V\rangle }_{{\rm{B}}}$	$\alpha {|+{{\rm{y}}}_{{\rm{S}}}\rangle }_{3}\pm i\beta {|-{{\rm{y}}}_{{\rm{S}}}\rangle }_{3}$	$|\uparrow \rangle \langle +{{\rm{y}}}_{{\rm{S}}}|\mp i|\downarrow \rangle \langle -{{\rm{y}}}_{{\rm{S}}}|$
	${|L\rangle }_{{\rm{A}}}{|{\pm }_{{\rm{S}}}\rangle }_{2}$	${|H\rangle }_{{\rm{B}}}$	$\alpha {|-{{\rm{y}}}_{{\rm{S}}}\rangle }_{3}\pm i\beta {|+{{\rm{y}}}_{{\rm{S}}}\rangle }_{3}$	$|\uparrow \rangle \langle -{{\rm{y}}}_{{\rm{S}}}|\mp i|\downarrow \rangle \langle +{{\rm{y}}}_{{\rm{S}}}|$
		${|V\rangle }_{{\rm{B}}}$	$\alpha {|-{{\rm{y}}}_{{\rm{S}}}\rangle }_{3}\mp i\beta {|+{{\rm{y}}}_{{\rm{S}}}\rangle }_{3}$	$|\uparrow \rangle \langle -{{\rm{y}}}_{{\rm{S}}}|\pm i|\downarrow \rangle \langle +{{\rm{y}}}_{{\rm{S}}}|$
${|{-}_{{\rm{S}}}\rangle }_{{\rm{1}}}$	${|R\rangle }_{{\rm{A}}}{|{\pm }_{{\rm{S}}}\rangle }_{2}$	${|H\rangle }_{{\rm{B}}}$	$\alpha {|-{{\rm{y}}}_{{\rm{S}}}\rangle }_{3}\mp i\beta {|+{{\rm{y}}}_{{\rm{S}}}\rangle }_{3}$	$|\uparrow \rangle \langle -{{\rm{y}}}_{{\rm{S}}}|\pm i|\downarrow \rangle \langle +{{\rm{y}}}_{{\rm{S}}}|$
		${|V\rangle }_{{\rm{B}}}$	$\alpha {|-{{\rm{y}}}_{{\rm{S}}}\rangle }_{3}\pm i\beta {|+{{\rm{y}}}_{{\rm{S}}}\rangle }_{3}$	$|\uparrow \rangle \langle -{{\rm{y}}}_{{\rm{S}}}|\mp i|\downarrow \rangle \langle +{{\rm{y}}}_{{\rm{S}}}|$
	${|L\rangle }_{{\rm{A}}}{|{\pm }_{{\rm{S}}}\rangle }_{2}$	${|H\rangle }_{{\rm{B}}}$	$\alpha {|+{{\rm{y}}}_{{\rm{S}}}\rangle }_{3}\pm i\beta {|-{{\rm{y}}}_{{\rm{S}}}\rangle }_{3}$	$|\uparrow \rangle \langle +{{\rm{y}}}_{{\rm{S}}}|\mp i|\downarrow \rangle \langle -{{\rm{y}}}_{{\rm{S}}}|$
		${|V\rangle }_{{\rm{B}}}$	$\alpha {|+{{\rm{y}}}_{{\rm{S}}}\rangle }_{3}\mp i\beta {|-{{\rm{y}}}_{{\rm{S}}}\rangle }_{3}$	$|\uparrow \rangle \langle +{{\rm{y}}}_{{\rm{S}}}|\pm i|\downarrow \rangle \langle -{{\rm{y}}}_{{\rm{S}}}|$

**Figure 6:**
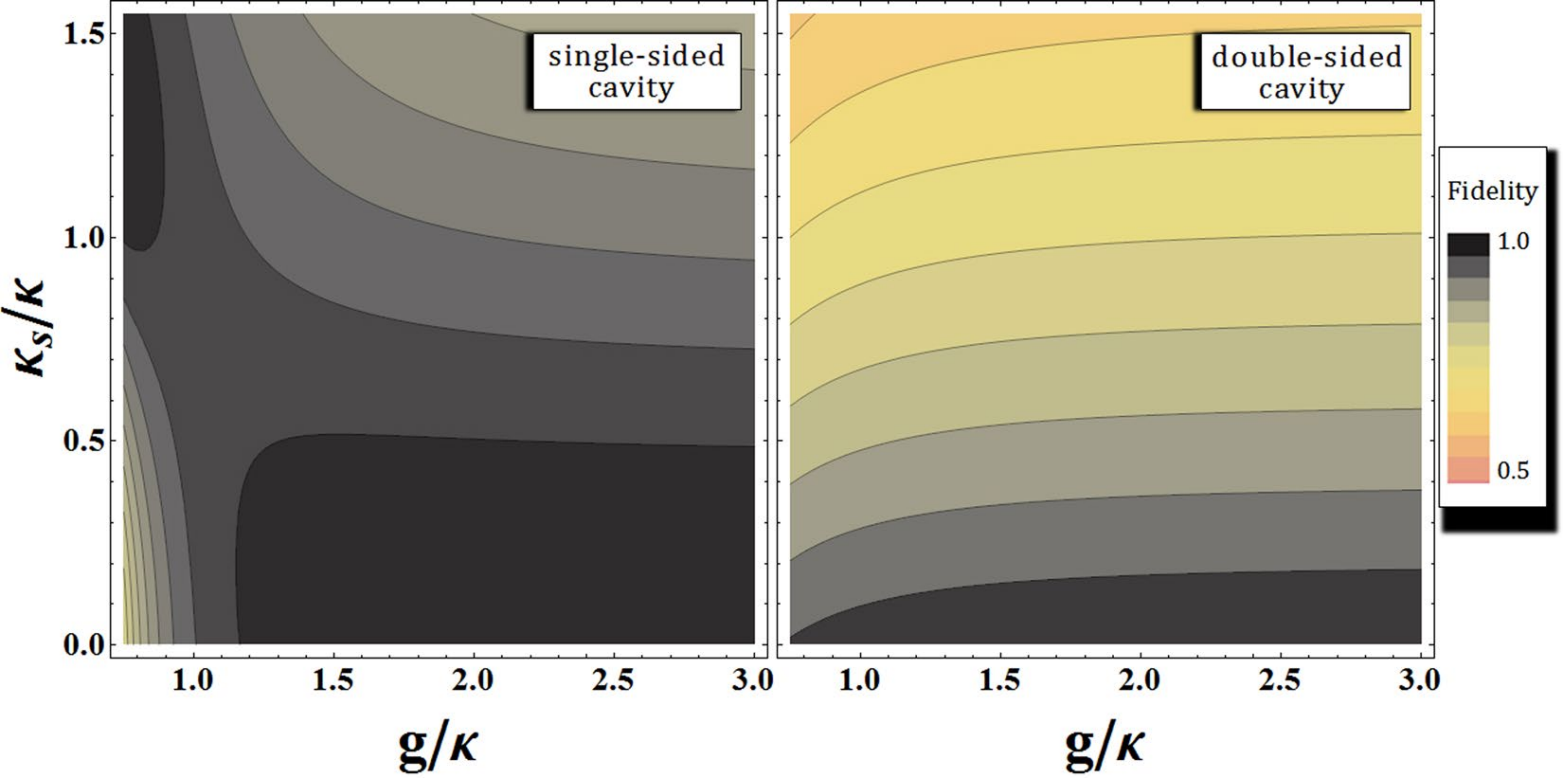
Plots represent fidelities F_S_ (QD inside a single-sided cavity) and F_D_ (QD inside a double-sided cavity) of the output states with respect to the differences in side leakage rate *κ*_*s*_/*κ* and the coupling strength *g*/*κ* between the QD and the cavity with fixed $\gamma /\kappa =0.1$ and ${\omega }_{{{\rm{X}}}^{-}}={\omega }_{c}$ (F_S_:$\omega -{\omega }_{c}=\kappa /2$, and F_D_: $\omega ={\omega }_{c}$). As described in the plots, *g*/*κ* should increase, and *κ*_*s*_/*κ* should decrease for ${{\rm{F}}}_{{\rm{S}}},{{\rm{F}}}_{{\rm{D}}}\to 1$ (black color range).

In our CQT scheme, the reliable performance of the QD-cavity systems is the central issue for the distribution of the authenticated entanglement channel, and for teleportation. So, we should calculate the fidelity of the interactions between photons and QDs inside single- and double-sided cavities to show the efficiency of the CQT scheme. Let us consider the ideal conditions of QDs (1 and 3) inside a single-sided cavity as $\omega -{\omega }_{c}=\kappa /2$ in Eq. 4, and of QD 2 inside a double-sided cavity as *ω* = *ω*_*c*_ in Eq. 9 with $g/\kappa =2.4$, $\gamma /\kappa =0.1$, *κ*_*s*_ = 0, and ${\omega }_{{{\rm{X}}}^{-}}={\omega }_{c}$ ^16,19–30^. As described in Eqs 4 and 9, we can obtain reflectances ($|{r}_{{\rm{0}}}(\omega )|=|{r}_{{\rm{h}}}(\omega )|\approx 1$) and phase shifts (${\phi }_{{\rm{rh}}}(\omega )=0$ and ${\phi }_{{\rm{r0}}}(\omega )=-\pi /2$), for QD1 and QD3 inside a single-sided cavity, and also the reflectances and the transmittances ($|{R}_{{\rm{h}}}(\omega )|=|{T}_{{\rm{0}}}(\omega )|\approx 1$ and $|{R}_{{\rm{0}}}(\omega )|=|{T}_{{\rm{h}}}(\omega )|\approx 0$), and phase shifts (${\phi }_{{\rm{R0}}}(\omega )={\phi }_{{\rm{Rh}}}(\omega )\approx 0$ and ${\phi }_{{\rm{T0}}}(\omega )={\phi }_{{\rm{Th}}}(\omega )\approx \pi$), for QD2 inside a double-sided cavity, under ideal conditions after the interactions of the QD-cavity system. Thus, the fidelities F_S_ of the QD inside a single-sided cavity (between the ideal state $|{\psi }_{{\rm{Id}}}^{{\rm{S}}}\rangle$ from Eq. 4 and the practical state $|{\psi }_{{\rm{\Pr }}}^{{\rm{S}}}\rangle$ from Eq. 3) and the fidelities F_D_ of the QD inside a double-sided cavity (between the ideal state $|{\varphi }_{{\rm{Id}}}^{{\rm{D}}}\rangle$ from Eq. 9 and the practical state $|{\varphi }_{{\rm{\Pr }}}^{{\rm{D}}}\rangle$ from Eq. 8) can be calculated as

15 $$\begin{array}{c}{{\rm{F}}}_{{\rm{S}}}=|\sqrt{\langle {\psi }_{{\rm{p}}{\rm{r}}}^{{\rm{S}}}|{\psi }_{{\rm{I}}{\rm{d}}}^{{\rm{S}}}\rangle \langle {\psi }_{{\rm{I}}{\rm{d}}}^{{\rm{S}}}|{\psi }_{{\rm{p}}{\rm{r}}}^{{\rm{S}}}\rangle }|=|\sqrt{[{|{r}_{{\rm{h}}}|}^{2}+{|{r}_{0}|}^{2}+2|{r}_{{\rm{h}}}||{r}_{0}|\sin ({\phi }_{{\rm{r}}{\rm{h}}}-{\phi }_{{\rm{r}}0})]/2({|{r}_{{\rm{h}}}|}^{2}+{|{r}_{0}|}^{2})}|,\\ {{\rm{F}}}_{{\rm{D}}}=|\sqrt{\langle {\varphi }_{{\rm{p}}{\rm{r}}}^{{\rm{D}}}|{\varphi }_{{\rm{I}}{\rm{d}}}^{{\rm{D}}}\rangle \langle {\varphi }_{{\rm{I}}{\rm{d}}}^{{\rm{D}}}|{\varphi }_{{\rm{p}}{\rm{r}}}^{{\rm{D}}}\rangle }|=|\sqrt{|{R}_{{\rm{h}}}+{T}_{{\rm{h}}}-{R}_{0}-{T}_{0}{|}^{2}/2({|{R}_{{\rm{h}}}|}^{2}+{|{R}_{0}|}^{2}+{|{T}_{{\rm{h}}}|}^{2}+{|{T}_{0}|}^{2})}|,\end{array}$$

where the input states are $(|R\rangle +|L\rangle )\otimes (|\uparrow \rangle +|\downarrow \rangle )/2$ (the QD-cavity system: single-sided) and $(|{R}^{\uparrow }\rangle +|{R}^{\downarrow }\rangle +|{L}^{\uparrow }\rangle +|{L}^{\downarrow }\rangle )\otimes (|\uparrow \rangle +|\downarrow \rangle )/2\sqrt{2}$ (the QD-cavity system: double-sided). As shown in Fig. 6, both fidelities F_S_ (the QD-cavity system: single-sided^16,19–22,24,30^) and F_D_ (the QD-cavity system: double-sided^23,25–29^) of the output states approach 1 when the coupling strength, *g*/*κ*, is strong $(g\gg (\kappa ,\gamma ))$, and *κ*_*s*_/*κ* is the small side leakage rate $(\kappa \gg {\kappa }_{s})$ with $\omega -{\omega }_{c}=\kappa /2$ (single-sided) and $\omega ={\omega }_{c}$ (double-sided). Table 2 summarizes the values of the fidelities (F_S_ and F_D_), Eq. 15, with regard to the differences in the side leakage rates (*κ*_*s*_/*κ*) for the fixed parameters as $g/\kappa =2.4$, $\gamma /\kappa =0.1$, and ${\omega }_{{{\rm{X}}}^{-}}={\omega }_{c}$ ^16,19–30^. We can obviously confirm acquiring reliable performance of the interactions between photons and the QD-cavity systems if side leakage rate *κ*_*s*_/*κ* is negligible, as shown in Table 2.

**Table 2:** The fidelities of the output states according to the differences in side leakage rate ${\kappa }_{s}/\kappa$ of the cavity for coupling strength $g/\kappa =2.4$, and the decay rate of exciton $\gamma /\kappa =0.1$, and ${\omega }_{{{\rm{X}}}^{-}}={\omega }_{c}$.

Fixed parameters	${{\boldsymbol{\kappa }}}_{{\bf{s}}}{\boldsymbol{/}}{\boldsymbol{\kappa }}$	${\bf{s}}{\bf{i}}{\bf{n}}{\bf{g}}{\bf{l}}{\bf{e}}{\boldsymbol{-}}{\bf{s}}{\bf{i}}{\bf{d}}{\bf{e}}{\bf{d}}({\boldsymbol{\omega }}{\boldsymbol{-}}{{\boldsymbol{\omega }}}_{{\bf{c}}}{\boldsymbol{=}}{\boldsymbol{\kappa }}/{\bf{2}})$	${\bf{d}}{\bf{o}}{\bf{u}}{\bf{b}}{\bf{l}}{\bf{e}}{\boldsymbol{-}}{\bf{s}}{\bf{i}}{\bf{d}}{\bf{e}}{\bf{d}}({\boldsymbol{\omega }}{\boldsymbol{-}}{{\boldsymbol{\omega }}}_{{\bf{c}}}{\boldsymbol{=}}{\bf{0}})$
		F_S_	F_D_
$$\begin{array}{c}g/\kappa =2.4\\ \gamma /\kappa =0.1\\ {\omega }_{{{\rm{X}}}^{-}}={\omega }_{c}\end{array}$$	1.5	**0.8721**	**0.6514**
	1.0	**0.9219**	**0.7503**
	0.5	**0.9746**	**0.8679**
	0.0	**0.9989**	**0.9956**

By adjusting the frequencies of the external field and cavity mode, we take the frequency detuning $\omega -{\omega }_{c}=\kappa /2$ ($\omega ={\omega }_{c}$) in QD inside a single (double)-sided cavity.

For our CQT scheme, the QD-cavity systems (single- and double-sided cavities) should be reliably performed during the interactions between photons and electrons. If the initial spin state (excess electron) in the QD-cavity system is $(|\uparrow \rangle +|\downarrow \rangle )/\sqrt{2}$, then this state will be a mixed state due to spin decoherence, $\tau \,(\tau \ll {{\rm{T}}}_{{\rm{1}}}^{{\rm{e}}})$, as follows:

16 $${\rho }(\tau )=\frac{1}{2}(\begin{array}{cc}1 & \exp (-\tau /{{\rm{T}}}_{{\rm{2}}}^{{\rm{e}}})\\ \exp (-\tau /{{\rm{T}}}_{{\rm{2}}}^{{\rm{e}}}) & 1\end{array}),$$

where ${{\rm{T}}}_{{\rm{2}}}^{{\rm{e}}}$ and ${{\rm{T}}}_{{\rm{1}}}^{{\rm{e}}}$ are the electron spin coherence time (~μs)^31–36^ and the electron spin relaxation time (~ms)^37–40^ in GaAs- or In(Ga)As-based charged QDs. Thus, if the interaction time between a photon and electron of a QD in our CQT scheme is much shorter than electron spin coherence time, ${{\rm{T}}}_{{\rm{2}}}^{{\rm{e}}}$, we can accomplish reliable performance of the CQT due to Eq. 16, $[\tau \ll {{\rm{T}}}_{{\rm{2}}}^{{\rm{e}}}\Rightarrow \exp (-\tau /{{\rm{T}}}_{{\rm{2}}}^{{\rm{e}}})\to 1]$. So the total time to realize the protocol (including propagation times) should be shorter than the spin coherence time, which is in the ns or μs range. Furthermore, we assume the photon bandwidth is much smaller than cavity broadening ($\omega -{\omega }_{c}\ll \kappa /2$), so the frequency detuning can be precisely set^21^. Also, we should consider two kinds of exciton dephasing (optical dephasing and spin dephasing) in the QD-cavity systems. Exciton dephasing reduces the fidelity by the amount of $[1-\exp (-t/{{\rm{T}}}_{{\rm{2}}}^{{\rm{e}}})]$ where *t* is the cavity photon life time. The optical dephasing can reduce the fidelity less than 10% due to extending the time scale of the excitons to hundreds of picoseconds^71,72^. The effect of the spin dephasing can be neglected because the spin decoherence time ($\tau  > 100\,{\rm{ns}}$) is several orders of magnitude longer than the cavity photon lifetime (typically $t < 10\,{\rm{ps}}$)^73,74^. In addition, when the CQT scheme is implemented with the QD-cavity systems in the optical fiber, for the path of the transferring photons, our scheme requires the interferometric stabilizations due to the fiber sensitivity to environmental temperature.

Consequently, we presented a CQT scheme consisting of Trent (the controller and provider of the entanglement channel), Alice, and Bob (both of them users) using the interactions of photons and QDs inside one double-sided^23,25–29^ and two single-sided^16,19–22,24,30^ optical cavities. Because our CQT scheme employs feasible QD-cavity systems, as mentioned above, we can experimentally acquire authenticated and controlled quantum teleportation.

## Conclusion

So far, our CQT scheme utilizes the interactions between flying photons and the located electrons of QDs inside cavities for the construction of an authenticated entanglement channel (electron spin 1 – photon A – photon B) and controlled teleportation of an unknown state (electron spin 2 → electron spin 3). The deterministic interactions of photons and the QD-cavity systems are the most critical ingredients for the utilized multi-qubit gates in our CQT scheme. Therefore, we should consider experimental implementation of the QD-cavity system in practice.

For high-performance (high-fidelity) in the interactions between the photons and the QD-cavity systems, we need to obtain strong coupling (*g*) and a small side leakage rate (*κ*_*s*_) between the QD and the single- and double-sided cavities. Bayer *et al*.^75^, for strong coupling, researched micropillars with diameter $d=1.5\,{\rm{\mu }}m$, and obtained $\gamma /2\approx 1\,{\rm{\mu }}\mathrm{eV}$ (decay rate of X^−^) when temperature $T\approx 2\,K$. The coupling strength in a micropillar cavity at $d=1.5\,{\rm{\mu }}m$ can be achieved $g/(\kappa +{\kappa }_{s})\approx 0.5$ for quality factor $Q\approx 8800$ ^19^, and can increase to $g/(\kappa +{\kappa }_{s})\approx 2.4$ for quality factor $Q\approx 40000$ ^76^. The side leakage rate *κ*_*s*_ depends on fabrication and various cavity details such as materials, structures, and size. When $g\approx 80\,{\rm{\mu }}\mathrm{eV}$ and quality factor $Q\approx 40000$ (including side leakage rate *κ*_*s*_) have been realized with ${{\rm{In}}}_{0.6}{{\rm{Ga}}}_{0.4}{\rm{As}}$, Reitzenstein *et al*.^20^ demonstrated that the side leakage (and unwanted absorption), *κ*_*s*_, can be made rather small by optimizing the etching process (or improving the sample growth), $g/(\kappa +{\kappa }_{s})\approx 2.4$. On the other hand, coupling strength *g* depends on QD exciton oscillator strength and mode volume *V*, while cavity field decay rate *κ* is determined by the cavity quality factor, and coupling strength *g* and cavity field decay rate *κ* can be controlled independently to achieve a larger $g/(\kappa +{\kappa }_{s})$. Loo *et al*.^77^ achieved $g\approx 16\,{\rm{\mu }}\mathrm{eV}$ and $\kappa \approx 20.5\,{\rm{\mu }}\mathrm{eV}$ with $Q\approx 65000$ when $d=7.3\,{\rm{\mu }}m$ for a micropillar. And the quality factor improved to $Q\approx 215000(\kappa \approx 6.2\,{\rm{\mu }}\mathrm{eV})$ with a smaller side leakage rate^78^.

Besides, the conditions of the QD-cavity system require a long electron spin coherence time, ${{\rm{T}}}_{{\rm{2}}}^{{\rm{e}}}$, and electron spin relaxation time, ${{\rm{T}}}_{{\rm{1}}}^{{\rm{e}}}$, and techniques for manipulation and preparation of the single electron spin for reliable interactions and suitable storage of the quantum state. Electron spin coherence time ${{\rm{T}}}_{{\rm{2}}}^{{\rm{e}}}$ can be extended to *μs* by suppressing the nuclear spin fluctuations^31–33^ or by using spin echo techniques^32,34–36,79^. Also, the decoherence time is theoretically predicted to be as long as the spin relaxation time, which is currently 20 ms at a magnetic field 4 T and at 1 K^38^ and can be much longer for a lower magnetic field^39,40^. Moreover, the interactions between a photon and the QD-cavity system in our scheme comply with the spin selection rule for spin-dependent optical transitions of X^−^. Thus, we should keep the low magnetic field, since the transitions, that $|\uparrow \rangle \to |\uparrow \downarrow \Uparrow \rangle \,(|\downarrow \rangle \to |\downarrow \uparrow \Downarrow \rangle )$ is driven by $|L\rangle \,(|R\rangle )$, of the optical cavity are almost identical in our scheme. Before the arrival of the flying photons, the users initialize their spins by optical pumping or optical cooling^44,62^, followed by single-spin rotations^31,40^. The time needed for the coherent control of electron spins has been suppressed into the scale of picosecond in the semiconductor quantum dot^45^.

As mentioned in the above experimental research, a charged QD (negatively charged exciton) inside single- and double-sided cavities (QD-cavity systems) is one of the promising components in our CQT scheme for the distribution of an authenticated entanglement channel and controlled teleportation. Specifically, we can achieve an experimentally realizable CQT scheme with high fidelity by employing QD-cavity systems. Our CQT scheme has advantages besides experimentally feasible implementation, as follows.

1. For distribution of the authenticated entanglement channel by Trent, and controlled teleportation between users, we designed our scheme using QD-cavity systems and can obtain high fidelity when the QD-cavity systems (single- and double-sided)^16,19–30^ are prepared under the experimental conditions (strong coupling strength and small side leakage rate, $g\gg (\kappa ,\gamma )$ and ${\kappa }_{s}\ll \kappa$), with current technology.
2. For maximization of the advantages of quantum sources, our CQT scheme employs flying photons and electrons in QDs inside microcavities. A flying photon is the best resource to communicate with fast and reliable manipulation, but it is inappropriate to store it for long time due to the increasing decoherence effect. An electron confined to QDs inside cavities can acquire a long coherence time for storage of the state due to long electron spin coherence time (${{\rm{T}}}_{{\rm{2}}}^{{\rm{e}}} \sim {\rm{\mu }}s$)^31–36^ within a limited spin relaxation time (${{\rm{T}}}_{{\rm{2}}}^{{\rm{e}}} \sim {\rm{ms}}$)^37–40^ in GaAs- or In(Ga)As-based charged QDs. Thus, the distribution of entanglement channels between Alice and Bob is constructed using two flying photons (with fast and reliable manipulation), and the authentication (Trent) and teleportation of the unknown state (Alice and Bob) utilize electrons in QDs inside microcavities (a long coherence time for storage) in our CQT scheme.
3. In our CQT scheme using QD-cavity systems, Trent simultaneously plays roles as the channel provider and the trust center for authentication of the entanglement channel. It is necessary to authenticate legitimate users operating the teleportation. Thus, our designed scheme can guarantee to certify a secure entanglement channel through Trent’s measurement result of electron spin 1.

Consequently, we demonstrated that our CQT scheme has the advantage of experimentally feasible realization using QD-cavity systems, and efficiency and security in terms of quantum communication.
